# Exploring Laser-Induced Plasma Spectroscopy for Skin Cancer Patients: A Preliminary Study

**DOI:** 10.3390/diagnostics15162116

**Published:** 2025-08-21

**Authors:** Dimitrios Sgouros, Emmanouil Karampinis, Melpomeni Theofili, Georgia Pappa, Panagiotis Theofilis, Sofia Theotokoglou, Anna Syrmali, Alexander Katoulis

**Affiliations:** 1 Second Department of Dermatology-Venereology, National and Kapodistrian University of Athens, Medical School, “Attikon” General University Hospital, 12462 Athens, Greece; gpappa100@gmail.com (G.P.); theotokoglousofia@gmail.com (S.T.); annasyrmali@gmail.com (A.S.); alexanderkatoulis@yahoo.co.uk (A.K.); 2 Second Dermatology Department, School of Health Sciences, Aristotle University of Thessaloniki, 54124 Thessaloniki, Greece; emankarampinis@gmail.com; 3 Department of Dermatology, Faculty of Medicine, School of Health Sciences, University General Hospital of Larissa, University of Thessaly, 41110 Larissa, Greece; 4 First Department of Dermatology-Venereology, National and Kapodistrian University of Athens, Medical School, “Andreas Syggros” Hospital, 16121 Athens, Greece; melpomenitheofili@hotmail.com; 5 1st Cardiology Department, Hippokration General Hospital, 11527 Athens, Greece; panos.theofilis@hotmail.com

**Keywords:** skin cancer, spectroscopy, melanoma, diagnosis, squamous cell carcinoma

## Abstract

Skin cancer is the most frequently diagnosed form of cancer worldwide. Diagnostic uncertainty can arise when macroscopic or dermoscopic evaluations do not clearly differentiate between benign and malignant lesions. Laser-induced plasma spectroscopy (LIPS), traditionally used in fields like materials science and environmental analysis, has recently gained attention for its applications in human tissue assessment. LIPS works by generating a (micro)plasma when a laser interacts with tissue, producing element-specific light emissions that can be analyzed in real time. In this study, we explored the potential of LIPS to differentiate between benign and malignant skin lesions using the Spectra-Scope^®^ Score (SSS). Our results revealed a clear distinction: benign lesions showed a median SSS of 1.7, while suspicious and malignant lesions had a significantly higher median score of 8.1 (*p* < 0.001). Receiver operating characteristic (ROC) curve analysis demonstrated strong diagnostic performance, with an area under the curve (AUC) of 0.82 (*p* < 0.001). The findings of this preliminary study support the high accuracy of LIPS in identifying malignancy and underscore its promise as a non-invasive, real-time diagnostic aid. Integrating SSS into clinical workflows could enhance the early detection of skin cancer and reduce reliance on invasive diagnostic procedures. However, further validation is needed to fully establish its role in routine dermatological practice.

## 1. Introduction

Skin cancer is the most common malignancy worldwide [1]. Although not all skin cancer types can metastasize, early diagnosis and timely intervention remain critical for improving patient outcomes. However, challenges can occur if the macroscopic or dermoscopic assessment fail to provide exact indicators of a specific benign or malignant lesion and the diagnosis of the specific skin lesion becomes challenging [2]. In those cases, histopathology examination becomes necessary to provide a definitive diagnosis. Apart from the initial visual inspection of the lesion and the last step of the histopathology study, can there be any other methods to give clues of the nature of the skin condition in question?

Extensive research has focused on developing innovative, non-invasive technologies for skin cancer detection, leveraging various imaging and analytical techniques. These include reflectance confocal microscopy, spectrophotometric intracutaneous analysis (SIAscopy), and multiphoton tomography [3]. More recently, laser-induced plasma spectroscopy (LIPS), or laser-induced breakdown spectroscopy (LIBS), has emerged as a promising tool for distinguishing malignant from benign tumors by analyzing the biochemical composition of skin tissues through ultrashort laser pulses, without tissue damage [4].

The LIPS method has been used mostly in material science, geology, and environmental monitoring, but more studies are expressing an interest in using LIPS methods in biology systems and human tissues. LIPS generates microplasma upon laser interaction with the tissue, emitting element-specific light that is analyzed spectroscopically in real time. Cancerous tissues typically show higher levels of calcium, magnesium, and phosphorus, reflecting increased cell turnover, abnormal proliferation, and metabolic dysregulation [4]. This finding was also reported in the study of El-Hussain et al., who found differences in calcium and magnesium spectral lines’ intensity in the LIBS spectra of non-neoplastic and malignant breast and colorectal tissue samples. Therefore, the question arises as to whether the LIPS technique can also have clinical implications in the case of skin cancer [5].

Skin cancer patients usually demonstrate systemic oxidative stress, systemic inflammation, and often hypercalcemia due to excessive cumulative UV radiation that has transformed cutaneous harmful changes into systemic consequences [6]. Skin cancer tissues, especially basal cell carcinoma (BCC) and squamous cell carcinoma (SCC), have been reported to present with high levels of reactive oxygen species (ROS) and altered local oxidative stress conditions. This disruption of redox balance can impair the immune system’s capacity to eliminate cancerous cells while also interfering with antioxidant defenses, apoptosis, and DNA repair mechanisms that normally protect against the initiation, promotion, and progression of cancer [7]. Elevated oxidative stress levels are often detected by specific transient receptor potential channels, which are associated with various physiological and pathological processes. Their activation frequently leads to a significant increase in intracellular calcium levels [8], and therefore, skin cancer tissues are anticipated to present with a higher load of calcium that can be recognized using the LIPS technique.

Therefore, our study aims to assess the use of the LIPS technique as a diagnostic tool in cases with suspicious or borderline lesions and prove its usefulness as a potential non-invasive diagnostic procedure for skin malignancy recognition. The primary endpoint of this pilot study is a diagnostic accuracy assessment of LIPS and its ability to distinguish malignant lesions from benign ones, and the secondary endpoints include determining the sensitivity and specificity of this method.

## 2. Materials and Methods

### 2.1. Study Design

**Figure 1:**
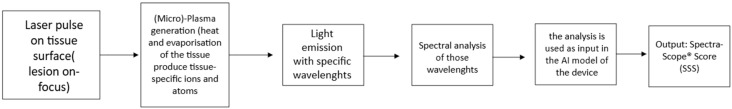
The flowchart of the LIPS technique used in the Spectra-Scope device.

This single-center study was designed as a preliminary evaluation of the diagnostic accuracy of the Spectra-Scope^®^ device (Speclipse, Sunnyvale, CA, USA), emphasizing its real-world applicability for skin tumor evaluation. Spectra-Scope^®^ is a compact, easy-to-carry device that analyzes the chemical composition of skin tissue using the LIPS method and provides practical scores that can be easily interpreted by clinicians/users. The LIPS method of the Spectra-Scope^®^ uses laser irradiation for a few nanoseconds to induce the formation of microplasma on the skin tissue without causing any tissue damage. The system then uses a deep neural network, trained on 5302 different light-emission readings, to help make accurate diagnoses. It is worth mentioning that the Spectra-Scope Score (SSS) does not have a definitive range of score that indicates tumor malignancy, as the provided score is based on a continuous spectrum of emission data rather than a binary classification (malignant/benign). The diagnostic interpretation depends on thresholds set through machine learning models according to the device’s model training [9] (Figure 1).

### 2.2. Study Participants

A total of 64 patients (Caucasian patients aged over 60 years old) with 67 skin tumors, including BCC, SCC, keratoacanthoma, Bowen’s disease, and melanoma, as well as benign tumors, were enrolled from the Dermato-Oncology Unit of the 2nd Department of Dermatology–Venereology at “Attikon” General University Hospital in Athens, Greece. Written informed consent was obtained from all participants. Initially, all lesions were evaluated clinically and dermoscopically by two experienced dermatologists (A.K. and D.S.). Each lesion was then assessed using the Spectra-Scope^®^ apparatus, and an SSS was recorded. Clinicians involved in the study were blinded to the SSSs during the diagnostic process, minimizing potential bias in clinical evaluation A total of 45 malignant lesions identified during clinical–dermoscopic examination were surgically excised for histopathologic confirmation. In the case of the 22 benign lesions, they were carefully monitored for six months without biopsy, ensuring comprehensive follow-up to rule out malignancy progression, and served as controls.

### 2.3. Statistical Analysis

Statistical analyses were conducted using SPSS software (version 25.0; SPSS Inc., Chicago, IL, USA). The Mann–Whitney U test determined between-group differences in continuous variables. The discriminative ability of the SSS in skin tumors was assessed through receiver operating characteristic (ROC) curve analysis with histopathology (malignant vs. benign) as the outcome and SSS as the continuous test variable. The area under the curve (AUC) was calculated as a measure of diagnostic accuracy. The optimal SSS cutoff point was determined by the Youden index. A *p*-value of <0.05 was considered statistically significant.

## 3. Results

**Figure 2:**
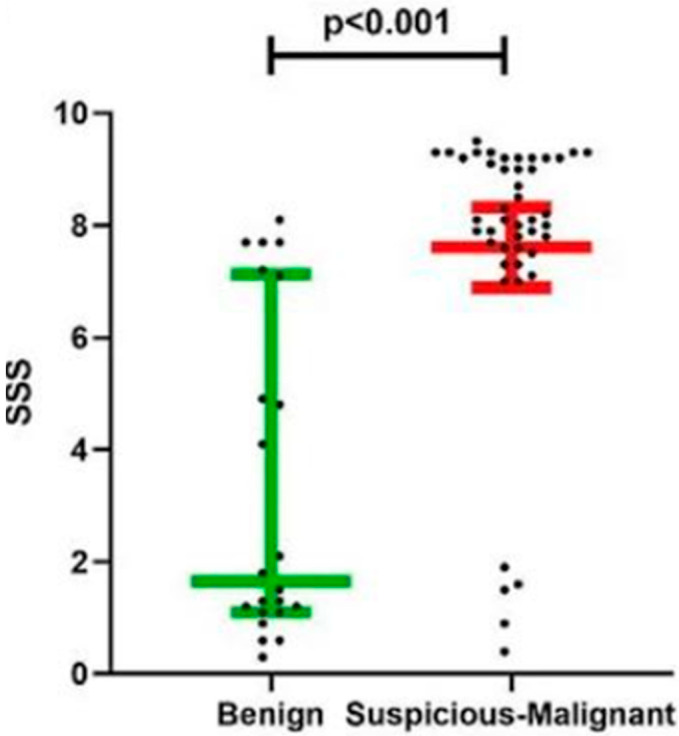
The role of the Spectra-Scope^®^ Score (SSS) in skin cancer. Scatter dot plot of SSS in clinico-dermoscopic benign and suspicious–malignant lesions.

Our findings demonstrated a different distinction between benign and malignant lesions based on the SSS. Benign lesions exhibited a median SSS of 1.7, whereas suspicious and malignant lesions showed a median SSS of 8.1 (*p* < 0.001), as illustrated in Figure 2. Histopathologic analysis corroborated these results, confirming the ability of SSS to reliably differentiate between benign and malignant lesions (Table 1).

Notably, malignant lesions exhibited distinct median SSS values when stratified by histological type, underscoring the versatility of LIPS in characterizing different tumor types. BCC and SCC had median SSS values of 8.0 and 8.7, respectively, while melanoma had the highest median SSS at 9.2 (Table 1). Finally, the lowest median SSS was reported in the case of Bowen’s disease. Although the sample size, particularly for benign lesions (n = 22), was limited, the use of Cliff’s Delta, which is a non-parametric measure of effect size, supports the robustness of our findings, with a large effect observed (Δ = −0.674), indicating a meaningful difference between groups.

**Figure 3:**
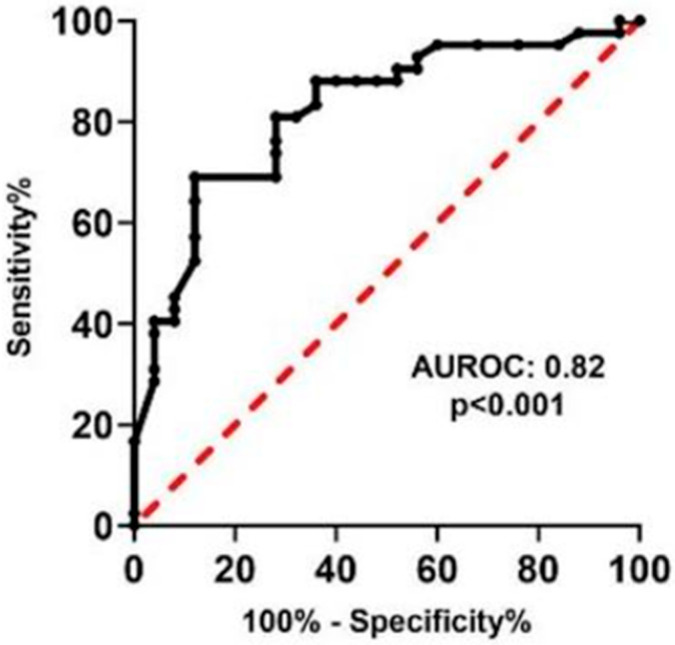
Receiver operating characteristic (ROC) curve of SSS in skin tumors.

ROC curve analysis confirmed the strong diagnostic performance of LIPS, with an area under the curve (AUC) of 0.82 (*p* < 0.001), as depicted in Figure 3. An optimal SSS cutoff of 7.3 achieved a sensitivity of 81% and specificity of 72%, demonstrating its reliability in distinguishing between benign and malignant skin lesions.

## 4. Discussion

Our study represents a pilot investigation that assesses LIPS as a valuable adjunct to existing diagnostic pathways. While histopathology remains the gold standard, LIPS has the potential to serve as a first-line screening tool, particularly for clinicians with limited dermoscopy expertise, including general practitioners and non-specialist physicians. Also, the LIPS approach can be of valuable importance in cases where the dermoscopy observation does not lead to an initial evaluation of a lesion, especially in ambiguous cases, and LIPS can be utilized as a complementary tool. By providing quantitative, reproducible data, LIPS minimizes the subjectivity of visual assessments and enhances early decision-making. Importantly, LIPS can optimize patient management by reducing unnecessary biopsies and specialist referrals without compromising diagnostic accuracy. These findings align with previous research [4,5], reinforcing its reproducibility and practical applicability in dermato-oncology. Compared to larger studies focusing on LIPS [4], this preliminary study confirms the value of spectroscopy methods in skin cancer detection and introduces SSS as a straightforward metric that may facilitate rapid clinical adoption and real-time decision-making.

The SSS does not have a fixed reference range specifically defining cancer, as it functions more as a relative indicator of the biochemical content—particularly the levels of calcium and magnesium—within the tissue being examined. Because of this, it may be clinically valuable to compare each lesion’s SSS to that of other lesions from the same patient, identifying any that fall significantly outside the expected range and marking them as potentially suspicious. When used in combination with dermoscopy or AI-assisted diagnostic tools, this comparative approach could improve diagnostic accuracy, helping to more effectively flag lesions with malignant potential [5].

Additionally, there are other studies that have evaluated the spectroscopy approach of skin cancer detection, such as elastic-scattering spectroscopy (ESS). In this study, the researchers used a device whose signal type for skin cancer assessment is light scattering. According to the theory behind ESS, the degree of variance among the cellular and subcellular components of a lesion can be determined based on photon backscatter reflections. Both ESS and SSS are not invasive and provide real-time feedback [10]. Because of their practical advantages, these methods can be particularly useful for evaluating suspicious lesions located in anatomical areas where performing a histopathological examination is difficult. In such cases, they may offer valuable insights into the potential malignancy of the lesion [11].

Another type of spectroscopy method used for skin cancer detection is electrical impedance spectroscopy (EIS). EIS is a non-invasive technique in which the clinician uses a device with a handheld probe and a disposable electrode that is placed directly on the skin. It works by measuring differences in electrical impedance to distinguish between healthy and potentially abnormal skin lesions. Electric impedance is defined as the resistance to the flow of the electric current. Different tissues resist the electric current differently, including benign and malignant skin tumors, due to their different cellular structures. One of the main limitations of this method is that seborrheic keratoses can be frequently inaccurately classified as malignant by EIS [12]. EIS also holds promise as a diagnostic biomarker and has the potential to be used in skin cancer detection and staging, as well as to monitor the effectiveness of treatment in individual patients and in therapeutic research. However, clear contraindications need to be established not only for the patients’ characteristics (patients with electronic implants, etc.) but also for the skin lesion characteristics (site of the lesion, presence of ulceration, and lesion size) [13]. The spectroscopy approaches differ in terms of equipment set-up, and the data provided offer insights into tissue structure, the SSS reveals biochemical composition, and EIS measures electrical resistance properties. Future studies combining those approaches may enhance the diagnostic accuracy of skin cancer detection and treatment.

As indicated by our results, the highest SSSs were reported in SCC and melanoma. This result can be explained by the cellular atypia, different metabolic profiles, and abnormal turnover of those cells, and the resulting changes in tissue composition further promote changes in calcium and magnesium levels that the Spectra-Scope tool detects through light emissions. Differences in melanin content and abnormal keratinization may contribute to the high SSSs. The presence of melanin could influence plasma energy transfer, altering how signals are emitted or detected, as in the case of LIPS. The histopathological features of the malignant lesion also play a vital role as, for example, in cases of necrosis due to intracellular Ca^(2+)^ overload, the SSS is anticipated to be higher [14]. In the cases of BCC, based on 25 lesions, the SSSs were also high (median score of 8). These high scores are possible attributed to structural changes, neovascularization (vessels that present with serum calcium and other elements that LIPS recognizes), and high local oxidative stress conditions. As this is an introductory study on the LIPS approach in skin lesions, more lesion-specific SSS studies are anticipated in the future, comparing, for example, SSSs between different types of BCCs such as pigmented or superficial vs. nodular, etc. [15].

Despite its promising results, this study has certain limitations. The single-center design and relatively small sample size may restrict the generalizability of our findings, and the absence of histopathologic confirmation for dermoscopically benign lesions may introduce diagnostic uncertainty. Also, the control/benign lesions were of a mixed nature (seborrheic keratosis, melanocytic nevi, etc.). However, the median score of 1.7 indicated that the lesions that do not present altered tissue biology structure or structural abnormalities can have low SPSSs, despite the heterogeneity of the lesions examined [16]. Also, all the patients were Caucasian, which also represents a limitation as skin structure and pigmentation differences across populations could potentially affect diagnostic performance and spectral readings as well as skin cancer subtypes and clinical presentations [17].

## 5. Conclusions

LIPS demonstrates high accuracy in distinguishing suspicious and malignant skin tumors from benign lesions, highlighting its potential as a non-invasive early diagnostic tool. Incorporating SSSs into clinical assessment strategies could revolutionize skin cancer detection, offering a patient-friendly alternative to traditional methods. Further research, including multicenter trials and cost-effectiveness analyses, is necessary to confirm its clinical utility and pave the way for the widespread adoption of LIPS technology.

## Figures and Tables

**Table 1:** Median Spectra-Scope^®^ Scores (SSSs) by histological type of skin lesion.

Histological Type of Lesion (N = 67)	Number of Cases (n)	Median SSS
Benign	22	1.7
Malignant lesions (total)	45	8.1
-Basal cell carcinoma	25	8
-Squamous cell carcinoma	7	8.7
-Bowen’s disease	2	4.85
-Keratoacanthoma	2	8.25
-Melanoma	9	9.2

## Data Availability

The data described in this study are available upon request from the corresponding author.
